# A Preliminary Study of Knowledge Transfer in Multi-Classification Using Gene Expression Programming

**DOI:** 10.3389/fnins.2019.01396

**Published:** 2020-01-17

**Authors:** Tingyang Wei, Jinghui Zhong

**Affiliations:** ^1^School of Computer Science and Engineering, South China University of Technology, Guangzhou, China; ^2^Sino-Singapore International Joint Research Institute, Guangzhou, China

**Keywords:** gene expression programming, evolutionary multitasking, classification, genetic programming, evolutionary computation

## Abstract

Gene Expression Programming (GEP), a variant of Genetic Programming (GP), is a well established technique for automatic generation of computer programs. Due to the flexible representation, GEP has long been concerned as a classification algorithm for various applications. Whereas, GEP cannot be extended to multi-classification directly, and thus is only capable of treating an *M*-classification task as *M* separate binary classifications without considering the inter-relationship among classes. Consequently, GEP-based multi-classifier may suffer from output conflict of various class labels, and the underlying conflict can probably lead to the degraded performance in multi-classification. This paper employs evolutionary multitasking optimization paradigm in an existing GEP-based multi-classification framework, so as to alleviate the output conflict of each separate binary GEP classifier. Therefore, several knowledge transfer strategies are implemented to enable the interation among the population of each separate binary task. Experimental results on 10 high-dimensional datasets indicate that knowledge transfer among separate binary classifiers can enhance multi-classification performance within the same computational budget.

## 1. Introduction

Classification is a fundamental and active research topic in data mining. Various real-world applications involving medical diagnosis, image categorization, credit approval, and etc., are covered by classification techniques. Formally, in a classification task, a classifier is to assign a class label *k* to the given input data *X*_*i*_ with features $X_{i}^{1}$, $X_{i}^{2}$, …, $X_{i}^{N}$ after being trained by data *X*_1_, *X*_2_, …, *X*_*M*_, where *N* and *M* represent the number of the features and the sample size, respectively. In this paper, we focus on the multi-classification problems in which the number of the candidate values for class labels is larger than two.

Generally, machine learning methods involving Neural Networks (Krizhevsky et al., 2012), Random Forests (Breiman, 2001), Support Vector Machine (Chang and Lin, 2011), and etc., are applied to solve the multi-classification problems. Considering the issue of the curse of dimensionality, many evolutionary algorithms (EA) have been utilized to assist aforementioned machine learning methods to tackle high-dimensional datasets, including Artificial Bee Colony (ABC) (Hancer et al., 2018), Particle Swarm Optimization (PSO) (Xue et al., 2012; Tran et al., 2018), and Genetic Programming (GP) (Chen et al., 2017). To be specific, these population-based algorithms can evolve individuals with a fitness function with respect to the machine learning classifier, and therefore can be conducted in either single-objective or multi-objective fashion. By searching effective feature subsets and limiting the subset size using EAs, the classifier can be trained in a more efficient way and the classification results can be more interpretable.

Unlike other population-based algorithms that must be implemented with a given machine learning classifier, GP is capable of completing both feature selection and classification independently owing to its tree structure. By converting the tree structure of GP into a string structure, Gene Expression Programming (GEP) (Zhong et al., 2017), a variant of GP, enjoys the same benefit as GP of independent classification ability with additional power of controlling bloat issue by restricted string length (Ferreira, 2002). With the automatic construction capability, GEP-based methods have emerged to show high effectiveness on symbolic regression (Cheng and Zhong, 2018; Huang et al., 2018; Zhong et al., 2018b), time series prediction (Zuo et al., 2004), knowledge discovery (Zhong et al., 2014), and etc.

Although GP and GEP can construct classification rules independently and have been prevailing in a plethora of applications involving spectral image categorization (Rauss et al., 2000), radar imagery recognition (Stanhope and Daida, 1998), medical diagnosis (Gray et al., 1996), credit approval (Sakprasat and Sinclair, 2007), and etc., they cannot be directly applied to multi-classification. To adapt GP and GEP to multi-classification, most researchers are devoted to manually configuring some contrived rules to achieve collision avoidance of class labels, thereby combining the results of multiple binary classifiers. In Muni et al. (2004), a novel evolutionary operator is designed to guide the population, and a meta-heuristic rule is supplied to iteratively remove output collision of different binary classifiers. To avoid output collision, the order, that the varying binary classifiers come into effect for prediction, can also be redesigned according to the accuracy and the reciprocal training samples (Zhou et al., 2003). Moreover, the well-established multi-objective techniques can also enhance the multi-classifiers by maintaining a pareto front of binary classifiers by considering precision, recall, and classification rule size, and employing negative voting to avoid output collision numerically (Nag and Pal, 2015). Notably, any individual in population of aforementioned GP and GEP can only be a binary classifier, hence it is still unnatural to extend these algorithms to multi-classification in despite of explorations in past few years. Furthermore, since nearly all the GP-based and GEP-based multi-classification methods straightforwardly depend on binary classifiers, it is fitness function and combining strategy of binary classifiers that relatively matter in the algorithmic design.

As discussed above, existing GP and GEP methods for multi-classification generally adopt contrived rules to avoid output collision of binary classifiers, and a crucial cause for output collision is the separate training process for each binary classifier, which potentially degrades the performance of multi-classifiers. In fact, intuitively, a classification rule trained by binary classifiers of one class can hopefully be utilized by another class as a rule component that can to some extent boost its own binary classification performance through recognizing the pattern of negative samples. According to the consideration above, this paper takes into account the Evolutionary Multitasking paradigm (Gupta et al., 2015, 2017; Ong and Gupta, 2016; Bali et al., 2019) to facilitate the multi-classification avoiding output collision of binary classifiers by enhancing the knowledge transfer among multiple binary classifiers. Equipped with the capability of latent genetic transfer, Evolutionary Multitasking can resolve many optimization problems simultaneously by enabling the knowledge transfer among different problems through the unified chromosome representation. In control of the synergies of searching space for varying optimization tasks (Gupta et al., 2016a,b; Da et al., 2018; Zhou et al., 2018), Evolutionary Multitasking, which can be easily employed on existing population-based algorithm (Feng et al., 2017; Chen et al., 2018; Liu et al., 2018; Zhong et al., 2019), have shown promising results on a vast number of cases in multi-objective optimization (Gupta et al., 2016c; Feng et al., 2018), symbolic regression (Zhong et al., 2018a), capacitated vehicle routing problems (Zhou et al., 2016), expensive optimization tasks (Min et al., 2017), and can be extended to a large scale version (Chen et al., 2019; Liaw and Ting, 2019) to enable some more scalable applications in the future. The methodology of Evolutionary Multitasking paradigm naturally fits the multi-classification problem, by treating each binary classification problem as an optimization task within certain function evaluations. Notably, concerning the multi-classification as Evolutionary Multitasking problem does not require a design for unified representation as the canonical Multifactorial Evolutionary Algorithm (MFEA) (Gupta et al., 2015) does, since each binary classification task (optimization task) in this scenario shares the same solution representation.

For canonical GP, knowledge transfer especially for Evolutionary Transfer Learning, has been widely investigated in past few years. Generally, two sorts of strategies prevails for knowledge transfer in canonical GP, modularization and initialization (O'Neill et al., 2017). For modularization, fitter canonical GP individuals in source domain can be evaluated and extracted as new function units in the GP population in the target domain (O'Neill et al., 2017), which eliminates the uncommon features between source domain and target domain. For initialization that is a really simple and direct way, GP individuals of higher fitness value in source domain and their subtrees often serve as the initial individuals and favorable components to select (Muller et al., 2019). Initialization techniques also include the knowledge transfer with respect to the feature importance. Using the ranks and fitness value of population in the source domain problems to vote for each feature, relatively fair feature importance can be obtained to guide the evolution of the target domain problems (Ardeh et al., 2019). Whereas, most relevant researchers have focused on the Evolutionary Transfer Learning, where one or several source problems are applied to assist the target problems, rather than the Evolutionary Multitasking, in which various problems are solved simultaneously with the same priority. Moreover, the existing works mainly rely on experiment design related to individual structure of canonical GP, so it is possible that the same strategies may not work in some variants of canonical GP. Therefore, as an important variant of canonical GP, GEP, with a string structure which is distinct from that of GP, should be investigated with some similar knowledge transfer techniques of Evolutionary Multitasking that is more general than Evolutionary Transfer Learning, for more potential promising possibilities. In this paper, GEP methods with different variation operators are employed with corresponding knowledge transfer techniques to show the effectiveness and the limits of the Evolutionary Multitasking methods in multi-classification, based on an existing multi-classification framework designed for GEP.

The rest of this article is organized as follows. Section 2 introduces a GEP-based multi-classification framework that the experimental study is based on. The canonical Evolutionary Multitasking paradigm, MFEA, is described briefly in section 3. The proposed knowledge transfer strategies are presented in section 4, followed by the experimental study in section 5. Eventually, the conclusions are drawn in section 6.

## 2. GEP Multi-Classification Framework

AccGEP (Zhou et al., 2003) is a well designed GEP-based algorithm for multi-classification. Hence, considering the prevalence and the maturity of this framework, this article will employ AccGEP to serve as the baseline method for the study of knowledge transfer. In this section, the basic concept and the algorithmic details of GEP will be presented, followed by the introduction of AccGEP.

### 2.1. GP and GEP

As a member of evolutionary algorithms, GP generally considers each solution for optimization problem as an individual of the whole population, in which the evolution of the algorithm is driven by variation operators encompassing mutation operators, crossover operators, and selection operators (Poli et al., 2008) among the individuals, like most meta-heuristic algorithms.

**Figure 1:**
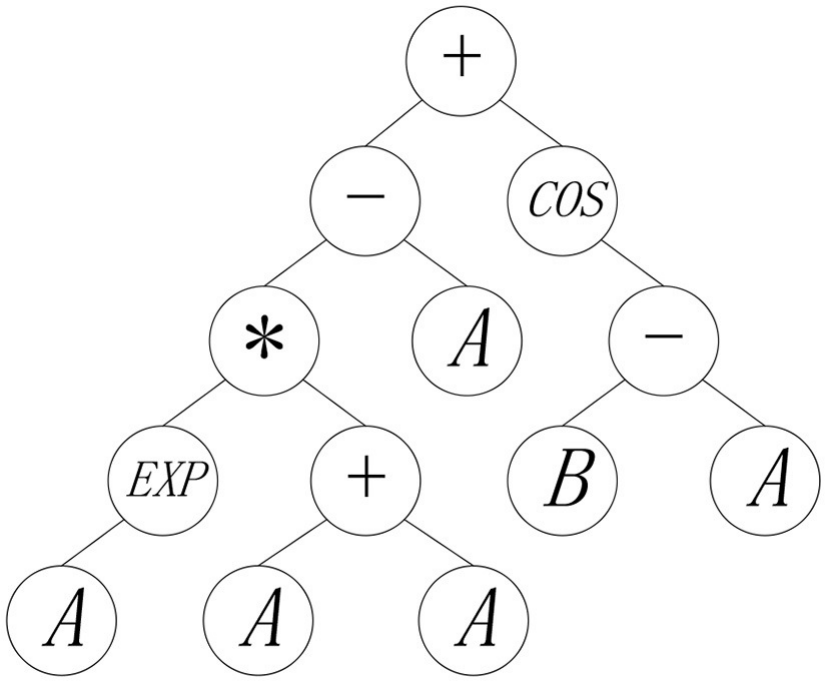
The encoding tree for mathematical expression.

Different from other population-based methods, the representation of each individual of GP is a mathematical expression encoded by a tree, where input variables are represented by leaf nodes, and the function operators like “−” and “*sin*,” are represented by intermediate nodes having offspring size of the same value with corresponding operands. For instance, Figure 1 depicts an individual that is encoded by mathematical expression, 2*A*exp(*A*) − *A* + cos(*B* − *A*), in GP population. For this mathematical expression, given the specific values of *A* and *B*, the output of the individual can be decoded in a bottom-up fashion to the root node of the representation tree. In canonical GP, the mutation, crossover, selection variation operators are applied to search for the more effective tree structures, thereby yielding the acceptable individuals with the satisfactory fitness values.

**Figure 2:**
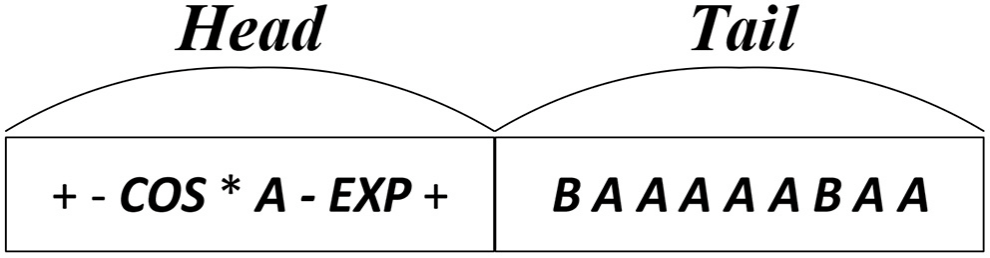
The encoding string for mathematical expression.

Distinct from canonical GP, GEP owns a string-based structure for each individual. Illustrating the same mathematical expression with the encoding tree of Figure 1, the string-based structure of GEP individual can be depicted as Figure 2, where the encoding tree is encoded by the string structure in a breadth-first-search traverse way. As illustrated by Figure 2, each individual of GEP population is composed of two parts, head part and tail part. In GEP, both the function units and terminal (i.e., variable) units constitute the head part of the string, while no function units but only the terminal units occur in tail part. During the evolution process of GEP, each string-based individual maintains a fixed length for both the head part and the tail part. Precisely speaking, a predefined constraint should be exerted on the length of head part(*h*) and the length of tail part(*l*) that:

(1) $$\begin{array}{l} l=h\cdot \left(u-1\right)+1 \end{array}$$

where *u* amounts to the maximum operand of the function unit, so as to guarantee that the encoded mathematical expression is complete (Poli et al., 2008). Furthermore, due to the breath-first-search traverse encoding mechanism, it is possible that some of the nodes saved in the string structure will not be utilized to encode mathematical expression.

### 2.2. AccGEP for Multi-Classification

With the capability of constructing mathematical expression, GEP-based algorithms is able to solve regression problems naturally, and can tackle binary classification issues by posing threshold values on regression tasks. For multi-classification problems, like most GEP-based classifiers, AccGEP, employed one-against-all (Aly, 2005) learning method, that is, treating an *M*-classification problem as *M* binary classification tasks. In one-against-all strategy, each binary classification problem is adopted to decide the data samples whether or not belong to a specific class, according to the fittest rule in the GEP population as follow:

(2) $$\begin{array}{l} \{\begin{array}{c} X_{i}\in Class_{j},GEP(X_{i})>0\\ X_{i}\notin Class_{j},GEP(X_{i})\leq 0 \end{array}. \end{array}$$

**Algorithm 1:** Covering Strategy

**Input:** *E*_+_ (*set of positive examples*), *E*_−_ (*set of negative examples*)
**Output:** *H* (*A set of GEP-based rules*)
1: /* *Initialization* */
2: *H* ← ∅
3: *L*_*min*_ ← +∞ (*minimum description length obtained*)
4: *L*_*H*_ ← 0 (*current description length*)
5: *L*_*theory*_ ← 0 (*theory bits*)
6:
7: /* *Learning* */
8: **Repeat**
9: Learn a rule *R* to cover the positive samples in *E*_+_
10: *E*_+_ ← *E*_+_−{s ∣ s can be covered by *R*}
11: /* *Pruning* */
12: *L*_*theory*_ ← *L*_*theory*_ + number of bits for encoding *R*
13: *L*_*exception*_(*H*) ←number of bits for encoding current exceptions
14: *L*_*H*_ ← 0.5·*L*_*theory*_ + *L*_*exception*_(*H*)
15: **If** (*L*_*H*_ < *L*_*min*_) **Then**
16: *H* ← *H*∪{*R*}
17: **Else**
18: Termination
19: /* *Update* */
20: **If** (*L*_*min*_ > *L*_*H*_) **Then**
21: *L*_*min*_ ← *L*_*H*_
22: **Until** *E*_+_ = = ∅

To deal with the complex feature spaces in multi-classification (Zhou et al., 2003), AccGEP applied the covering strategy to learn multiple rules for each binary classification problem. As shown in the algorithm 1, for each binary classification issue, AccGEP is designed to exploit a rule set that can cover all the positive data samples, and each rule in the rule set should be learnt by GEP and the according positive sample set with some criteria in each iteration. To be specific, the fitness function of each rule is designed as follow:

(3) $$\begin{array}{l} Fitness(R)=\{\begin{array}{c} \;0,Pre<0\\ Pre\cdot \exp(Rec-1),Pre\geq 0 \end{array}. \end{array}$$

where *Pre*, *Rec* represent the precision and recall in binary classification, respectively. Generally, *Pre* is computed as the ratio of true positive samples and predicted positive samples, while *Rec* is computed as the ratio of true positive samples and all positive samples. However, since the positive sample set, *E*_+_ in algorithm 1, shrinks in each iteration, a new formula for computing *Pre* is presented in AccGEP to better take advantage of the distribution information:

(4) $$\begin{array}{l} Pre=\left(\frac{TP}{TP+FP}-\frac{P}{P+N}\right)\cdot \frac{P+N}{N} \end{array}$$

where *TP*, *FP*, *P*, *N*, stand for the number of true positive samples, false positive samples, all positive samples in training set, all negative samples in training set of binary classification, correspondingly.

In order to allay the structural risks, the minimum description length principle in information theory is employed as a pruning technique for early stopping. As indicated in algorithm 1, *L*(*H*) stands for the description length of the current rule set, *H*. The learning process is terminated when the description length of rule set no longer declines. Moreover, *L*_*exception*_ and *L*_*theory*_ amount to the bits for encoding the error of the rule set, and the bits for encoding the rule set itself. The computation formula of the two description length are defined as follow:

(5) $$\begin{array}{l} \{\begin{array}{c} L_{exception}(H)=\log_{2}(C_{TP+FP}^{FP})+\log_{2}(C_{TN+FN}^{FN})\\ L_{theory}(H)=\log_{2}(N_{c})\sum_{i=1}^{s}L(R_{i}) \end{array}. \end{array}$$

where *TP*, *FP*, *TN*, *FN*, *N*_*c*_, *s*, *L*(*R*_*i*_), represent true positive samples, false positive samples, true negative samples, false negative samples, the number of distinct symbols applied in GEP, the number of the current rules, the valid length of individual for rule *R*_*i*_, accordingly.

**Figure 3:**
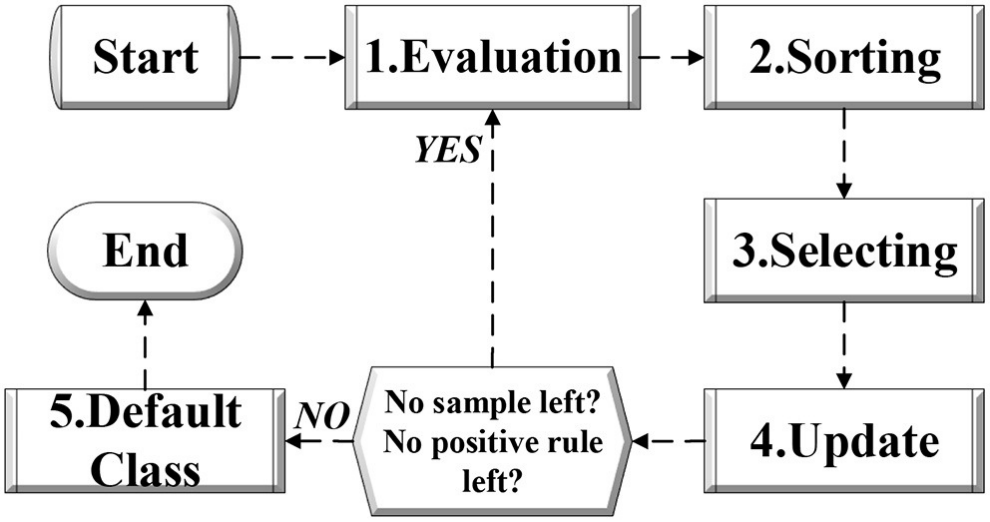
The flow chart for post pruning of AccGEP framework.

Having obtained multiple decision rules for each binary classification, a post-pruning technique is employed to combine the rules to yield the final results of multi-classification. Generally, as depicted in Figure 3 the combining strategy consists of steps as follow:

- *Evaluation*: In evaluation process, all the active rules in the rule set should be evaluated according to the fitness function as well as the existing samples in the training set.
- *Sorting*: In sorting process, all the active rules in the rule set should be sorted based on the fitness values.
- *Selecting*: In selecting process, the rule with the highest fitness value is selected, then it is moved into an ordered rule set. For the original rule set, the selected rule is removed.
- *Update*: In updating process, all the samples covered by the selected rule in selecting process will be removed as well.
- *Default Class*: With remaining samples and remaining rules that are able to cover any sample, AccGEP will proceed with the cycle from step 1 to step 4 as illustrated in Figure 3. Otherwise, the iteration will terminate and a default class label is decided, so as to avoid the scenario when all the rules will reject a new example. In general, the default class label will be set as the one that has most samples in the remaining sample set at the end of the algorithm cycle introduced above. Nevertheless, when there is a tie in the sample count in the remaining sample set or the remaining sample set is empty, the default class label can be determined randomly.

Through post-pruning process, AccGEP can attain an ordered rule set as well as a default class label. Subsequently, in the prediction phase for testing data, each testing sample belonging can be determined by the first rule that covers it in the ordered rule set. If a testing sample is rejected by all the rules, then the default class label will be assigned.

## 3. Multifactorial Evolutionary Algorithm

Inspired by the bio-cultural multifactorial inheritance, MFEA (Gupta et al., 2015), a typical Evolutionary Multitasking algorithm, is designed to fully exploit the potential of population-based algorithm to solve several optimization issues simultaneously. By introducing variables including factorial rank *r*, skill factor τ, scalar fitness ϕ, MFEA can enable the knowledge transfer among varying problems through a unified solution representation. Initially, all the initial solutions in the population should be evaluated across all the target problems. Subsequently, each individual will be assigned with a skill factor τ to indicate the task in which it has the most promising result. At length, the skill factor τ is determined by the factorial rank *r* of an individual across all the tasks as τ = arg_*j*_ min(*r*_*j*_), and then the scalar fitness ϕ can be computed accordingly by $\phi =\frac{1}{r_{\tau }}$. In order to improve the algorithm efficiency, in the subsequent evolution process, each individual will be only evaluated for the optimization task of its skill factor. By enabling the associative mating (Gupta et al., 2015), the skill factor of a certain individual can possibly undergo the variation.

With the techniques of assortative mating and selective evaluation for knowledge transfer, MFEA basically can comply with the similar work flow with the conventional Evolutionary Algorithms. In general, the main steps of MFEA can be illustrated as follow:

- *Initialization*: To start with, an initial population, *P*, is produced in MFEA. Then, all the individuals should be evaluated under all the problems, thereby getting the corresponding τ, ϕ, *r*.
- *Assortative Mating*: In each generation, the offspring will be generated through the conventional genetic operators including mutation and crossover. In MFEA, a control parameter, *rmp*, is applied to indicate the probability of the crossover between two individuals of different skill factor τ, which is concerned as a process of knowledge transfer. Otherwise, the crossover for parents of the same τ, or the mutation upon a single parent, is implemented.
- *Selective Evaluation*: Having generated an offspring population *O*, those individuals that undertake the crossover of different skill factor have undetermined τ. Intuitively, the skill factor of an individual should be set randomly based on the values of its parent. For those offsprings that merely undergo the casual crossover or mutation operator, skill factor will simply imitate their parents, known as a cultural transmission process (Gupta et al., 2015). Subsequently, the whole population will only be evaluated according to their best tasks. Aside from the optimization task τ, the fitness value of an individual for other problems should be assigned with ∞, in order that the true factorial ranks *r* would not be affected.
- *Population Update*: At the end of each generation, the skill factors and the scalar fitness of hybrid population *O*∪*P* should be re-evaluated to maintain only the individuals owning the best scalar fitness.

As discussed in section 1, an essential trigger for output collision in multi-classification is the separate training process of each binary classifier. Intuitively, binary classifier for different class labels might share some common structures and even some influential features. Based on the consideration above, it is believed that the latent genetic transfer attribute in Evolutionary Multitasking can enhance the performance of the existing GEP-based classifier by enabling the interaction among binary classifiers.

## 4. Proposed Algorithm

In this section, an Evolutionary-Multitasking-based classification method using GEP (EMC-GEP) is proposed. First, the general framework of the algorithm architecture is given. Then different knowledge transfer strategies for distinct GEP variation operators are discussed in section 4.2.

### 4.1. Framework

**Figure 4:**
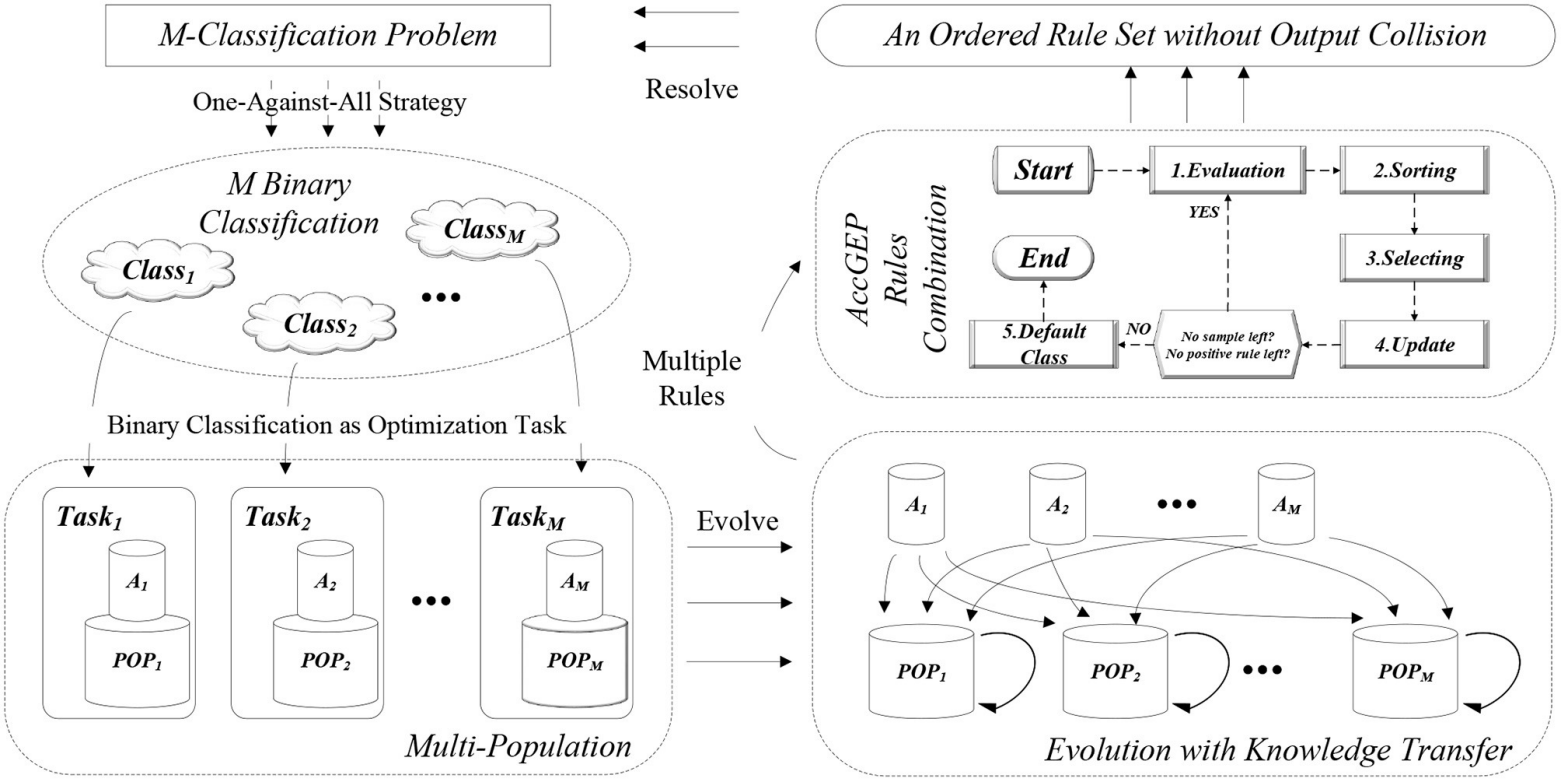
The general flow chart for the EMC-GEP framework.

As portrayed by Figure 4, the whole algorithm can be divided into four sections. First, the *M*-classification problem is degraded as *M* binary classification through One-Against-All learning. Then, each binary classification will be concerned as an optimization task that is tackled by each subpopulation, *POP*, that owns an archive, *A*. During the iterative evolution, all the subpopulation will undergo the variation operator as well as knowledge transfer. As depicted in Figure 4, the evolution process of each subpopulation include three parts: evolution within own subpopulation, knowledge transfer from its own archive, and knowledge transfer from the other archives. Notably, after each evolution iteration, the archive of each subpopulation will be updated as well. After the evolution process, the whole population can obtain various classification rules for each binary classification problem. With these learnt classification rules, the rules combination process (i.e., the post-pruning process depicted in section 2 and Figure 3), can combine all the binary rules to yield an ordered rule set (as well as a default class label as explained in section 2), so as to resolve the *M*-classification problem eventually. Each section will be detailed as follows.

#### 4.1.1. One-Against-All Strategy

One-Against-All is a casual strategy that treats the *M*-classification as *M* separate binary classification problems. As a variant of Error-Coding Output Codes (ECOC) (Dietterich and Bakiri, 1994), One-Against-All strategy is computationally efficient, compared with other ECOC-based strategies. Based on One-Against-All strategy, EMC-GEP will learn multiple rules for each binary classification problem, which is the same as the covering strategy of AccGEP as algorithm 1, thereby enhancing the robustness and stability of the classification framework.

#### 4.1.2. Paradigm in Multi-Population

In this article, the MFEA paradigm is implemented in a multi-population fashion as illustrated in Figure 4, with each subpopulation focusing on one optimization task. Since the canonical paradigm simply evolves the population as a whole encompassing all the target tasks and only one individual is reserved for each task in each iteration, it is possible that the collected information for each task is scant to guide the population to evolve. Moreover, the original framework of MFEA have only one control parameter *rmp* (Gupta et al., 2015) to enable the assortative mating for individuals of the unified representation, but such a framework cannot facilitate more flexible and extensible operation based on the population. Hence, based on discussion of Chen et al. (2018), Gong et al. (2019), and Liu et al. (2018), multi-population mechanism is employed to improve the stability of the MFEA paradigm and to enable more flexible operation on both sub-population and mixed-population (Chen et al., 2018).

For each task, a population, *POP*, is maintained along with an archive, *A*. Population, *POP*, is maintained to enable the flexible population-based operations and variation operators. Archive, *A*, is used to record some successful individuals of the corresponding *POP*, in order that those successful individuals hopefully can transfer their valuable solution components to its own *POP* or other *POP* in later knowledge transfer phase. Similar with the *POP*, each archive *A* will be updated according to the reciprocal subpopulation. Initially, each archive *A* should be initialized randomly. Then, after each evolution iteration, the individuals in population and the individuals in archive will be both sorted. With certain archive replacement probability, *arp*, the individuals in archive will be replaced by some individuals in population. Notably, the archive size is strictly smaller than that of population. Hence, with larger *arp*, the archive tends to resemble the fittest individuals in current population, while with smaller *arp*, more successful individuals in the searching history can be recorded, thereby enhancing the diversity of the archive individuals. Specifically, the archive update mechanism is illustrated as algorithm 2. It is notable that the fittest individual for each population may not be stored in the archive. The rationale behind this idea is intuitive. Generally, the archive is used to update two sorts of populations, its own population and other populations. To update its own population, the fittest individual is not necessarily stored in the archive since the self population expects more randomness and history information from the archive. To update other populations, although the fittest individual may own the most useful information in its own problem context, the synergies between the source archive and target population is uncertain. Therefore, we apply a loosely organized update archive to store both the good individuals and historical individuals to provide a more comprehensive transfer for other populations.

**Algorithm 2:** Archive Update

**Input:** *POP*_*i*_, *A*_*i*_, *arp*, *n*(*size of* *A*_*i*_)
**Output:** *A*_*i*_
1: /* *Sorting* */
2: Sorting population *POP*_*i*_ according to the fitness value
3: Sorting archive *A*_*i*_ according to the fitness value
4:
5: /* *Update* */
6: *j* ← 0
7: **For** *j* < *n* **Do**
8: **If** (*rand*(0, 1) < *arp*) **Then**
9: *A*_*i, j*_ ← *POP*_*i, j*_
10: (*A*_*i, j*_, *POP*_*i, j*_ *amount to the* *j-th individual in* *A*_*i*_, *POP*_*i*_)
11: *j* ← *j*+1
12: **End For**
13: **Return** *A*_*i*_

#### 4.1.3. Evolution Process

Distinct from previous works on knowledge transfer in multi-population (Chen et al., 2018; Liu et al., 2018), where the evolutionary operator is directly employed on two different populations, this paper utilizes the archive as the group of representative individuals of each population for knowledge transfer. As depicted in Figure 4, the evolution process of EMC-GEP involve three sections, self evolution (i.e., *POP*_*i*_ ← *POP*_*i*_), self transfer (i.e., *POP*_*i*_ ← *A*_*i*_), cross transfer (i.e., *POP*_*i*_ ← *A*_*j*_). The reason why self transfer is adopted in this paper is that, some useful solution components may not be fully exploited and may be forgotten by the subpopulation. Therefore, it is believed that the knowledge transfer from the “former” subpopulation toward the current subpopulation may help as cross knowledge transfer.

Generally, MFEA paradigm employs a probability variable *rmp* to control the mutual knowledge transfer for individuals of distinct skill factors (Gupta et al., 2015). Whereas, in this paper, a step-wise transfer control mechanism is applied to enable a more stable knowledge transfer process like (Da et al., 2018). As illustrated in algorithm 3, the transfer process is launched whenever the iteration count *t* can be divided by a certain transfer interval δ. Unlike those methods that try to adaptively select a similar task to transfer (Chen et al., 2019), as a preliminary study, this paper simply randomly selects an archive *A*_*j*_ for each subpopulation *POP*_*i*_, where *i* may not necessarily differ from *j* due to the discussion above.

It is notable that, same with covering strategy of AccGEP in algorithm 1, EMC-GEP also learns multiple rules for each binary classification task, and consistently the number of each binary classfication rules for distinct tasks can be different. Hence, it is possible that some binary classification tasks are still searching for the rules to cover the positive samples, while other tasks may already terminate. In this special circumstance, those archives, of which the reciprocal population's learning process has terminated, will still remain for knowledge transfer of those active population, and will undergo no changes during the evolution.

**Algorithm 3:** Evolution with Knowledge Transfer

**Input:** *POP*_1_, *POP*_2_, …, *POP*_*M*_, *A*_1_, *A*_2_, …, *A*_*M*_, δ (*transfer interval*)
**Output:** *New Population and New Archives*
/* *Preparation* */
*t* ← 1
Generate initial *M* population randomly.
Initialize *M* archive with corresponding population.
/* *Evolution* */
**While** *ending condition not satisfied* **Do**
/* *Searching* */
**For** *each subpopulation* *POP*_*i*_ **Do**
**If** *t % δ* = = 0 **Then**
/* *Transfer* */
*k* ← *rand*(1, *M*)
*POP*_*i*_ ← Transfer(*POP*_*i*_, *A*_*k*_)
**Else**
Self Evolution
**End For**
/* *Updating* */
**For** *each archive* *A*_*i*_ **Do**
*A*_*i*_ ← Update(*POP*_*i*_, *A*_*i*_) as Algorithm 2
**End For**
*t* ← *t* + 1
**End While**

#### 4.1.4. Rules Combination Using AccGEP

After the evolution process for those population aiming at varying binary classification tasks, we can obtain a vast number of classification rules, among which multiple rules are utilized for the same binary classification issue. To avoid output conflict, a combination phase is necessary for analyzing these rules. In this paper, EMC-GEP will adopt the same strategy as the post-pruning phase in AccGEP, which has already been specifically explained in Figure 3 and section 2.2.

### 4.2. Knowledge Transfer

The knowledge transfer has been investigated in various population-based algorithms, and the investigation mainly concentrated on the chromosome representation (Zhou et al., 2016; Zhong et al., 2018a), and the problem similarity (Da et al., 2018; Chen et al., 2019). However, in this paper, the problem representation for each binary classification problem does not require redesign, and we tend to select the archive randomly to assist the target task. The vital concern of our study is that, most knowledge transfer research highly depends on the data structure, and the efforts on GEP-based method are insufficient to supply a brief understanding of knowledge transfer effect on GEP. Hence, to add to a preliminary insight, this paper tries to employ knowledge transfer operations on different evolutionary operators in GEP.

#### 4.2.1. GEP With Canonical Variation Operator

Originally, the variation operators of GEP include mutation operator and crossover operator (and sometimes rotation operator) based on the string structure in Figure 2. Considering the data structure, the variation operator that really matters in knowledge extraction of the canonical GEP is crossover operator, since crossover operation can extract a continuous segment of an individual, and it is believed that the continuous segment can serve as useful genetic material for some classification problems.

To be specific, in GEP, crossover operator involves two operations, single-point crossover and two-point crossover. For single-point crossover, the crossover operation of GEP individuals resemble the behavior of Genetic Algorithm. Due to the breath-first-search encoding style of GEP, the forward part of crossover can serve as a skeleton of an expression tree. Taking the expression tree in Figure 1 as an example, the first four operators, “+,” “−,” “cos,” “*,” in a combination as first four nodes in a string-based individual, can construct a basic skeleton of the whole mathematical expression, which can be regarded as a form of transferable knowledge. For two-point crossover, the skeleton of an expression tree can also be extracted in the same way as one-point crossover. Moreover, with more segmented structure, the two-point crossover can hopefully extract the useful structure of an individual more flexible by enabling cutting out the intermediate string section of GEP individual.

To achieve the knowledge transfer through crossover operator, whenever knowledge transfer is launched in algorithm 3, the two parents of a crossover operator should be selected in target population *POP*_*i*_ and the source archive *A*_*k*_ separately. Aside from the selection choice, the crossover operation remains unchanged in other respects.

#### 4.2.2. GEP With DE-Based Variation Operator

Besides the conventional evolutionary operators, some variants of GEP methods can employ DE-based operators by transforming the string construction process into a continuous optimization method, which is highly extensible and has shown promising capability in applications like symbolic regression (Zhong et al., 2015).

In general, individuals in Differential Evolution (DE) (Storn and Price, 1997) should undergo mutation operation, where each element in individual will be replaced, in certain probability, by some random element added to a scaled difference element (Storn and Price, 1997). There are various mutation strategies frequently applied in the literature involving “DE/rand/1,” “DE/current-to-best/1,” “DE/best/1,” In this paper, as in Zhong et al. (2015), “DE/current-to-best/1” is employed as defined follow:

(6) $$\begin{array}{l} v_{i,g}=x_{i,g}+F_{i}\cdot \left(x_{best,g}-x_{i,g}\right)+F_{i}\cdot \left(x_{r_{1},g}-x_{r_{2},g}\right) \end{array}$$

where *v*, *x*, *F*, *i*, *g*, *r*_1_, *r*_2_, stand for new element, original element, mutation control parameter, individual index, dimension index, the first random index, and the second random index, accordingly. To apply the DE-based operator in GEP, SLGEP (Zhong et al., 2015) can transform the difference operation in equation 6 into a matching binary operator as:

(7) $$\begin{array}{l} \psi (a,b)=\;\{\begin{array}{c} 1,a\neq b\\ 0,a=b \end{array}. \end{array}$$

Then subsequently, the mutation operation of DE in equation 6 can be changed into a probability computation process:

(8) $$\begin{array}{l} \phi =1-\left(1-F\cdot \psi \left(x_{best,j},x_{i,j}\right)\right)*\left(1-F\cdot \psi \left(x_{r_{1},j},x_{r_{2},j}\right)\right) \end{array}$$

where the probability ϕ is adopted to control mutation operation of a specific node on position *j* in string structure representation in Figure 2. That is, when a random value in [0,1] is smaller than corresponding ϕ, then the node in the reciprocal position should be replaced by a newly sampled node, where the new node is sampled by the frequency record of all the nodes in the population as Zhong et al. (2015). The evolution process in SLGEP can be conluded as algorithm 4.

**Algorithm 4:** Evolution Process of SLGEP

**Input:** *F, r*_1_, *r*_2_, *x*_1_, *x*_2_, …, *x*_*M*_, *CR* (*replacement probability*), *k*(mandatory mutation index)
**Output:** *New Population*
**For** *each individual* *x*_*i*_ **Do**
/* *Variation* */
**For** *each dimension* *x*_*i, j*_ **Do**
Compute probability ϕ based on equation 8
**If** (*rand*_1_(0,1) < *CR* OR *j* = = *k*) AND *rand*_2_(0,1) < ϕ **Then**
*u*_*i,j*_ ← “Frequency-based Assignment” (Zhong et al., 2015)
**Else**
*u*_*i,j*_ ← *x*_*i, j*_
**End For**
/* *Selection* */
**If** *f*(*u*_*i*_) < *f*(*x*_*i*_) **Then**
*x*_*i*_ ← *u*_*i*_
**End For**

To achieve knowledge transfer based on the DE-based operator in SLGEP, similar to the strategy for canonical operator, this paper simply selects the individuals in archive to complete the computation process in the DE-based operator. The core computation part in DE-based operator is equation 8. According to the transfer paradigm Transfer (*POP*_*i*_, *A*_*k*_) in algorithm 3, for computation of ϕ, *x*_*best, j*_, *x*_*r*_1_, *j*_, *x*_*r*_2_, *j*_ are selected from external archive *A*_*k*_, and *x*_*i, j*_ is selected from population *POP*_*i*_. Notably, the “Frequency-based Assignment” in the original work is grounded on the frequency record of each sort of node in the whole population, which can also concerned as a form of useful knowledge especially for feature selection. Resembling the feature-wise knowledge transfer in Ardeh et al. (2019), this paper also enables the transfer of the node frequency by applying the frequency record of the archive *A*_*k*_ upon the individual assignment in population *POP*_*i*_.

## 5. Experimental Study

To verify the assumption that the proposed techniques can hopefully allay the conflict of each binary classification problem, the comparative studies on 10 high-dimensional multi-classification datasets for distinct GEP operators with their according transfer strategy are conducted. Aside from the direct comparative results, a relatively detailed discussion is also provided for a deeper insight on the effectiveness of knowledge transfer from various “source archives.” The comparison among the proposed method, *K* Nearest Neighbor, and Decision Tree is also provided. For all the experimental studies, the results are yielded by 30 independent trials, and the Wilcoxon sign-rank test (Wilcoxon, 1992) with α = 0.1 is performed to check for the significant difference of the experiment results.

### 5.1. Parameter Settings

Nearly all the fundamental settings of EMC-GEP are based on the original recommended settings of AccGEP in Zhou et al. (2003). In detail, the function set includes {+, -, *, /, **Sqrt**, **IF**}. The terminal set totally depends on the given classification problems, in addition to a list of constants, {1, 2, 3, 5, 7}. As for the algorithmic parameters, the chromosome length, the population size and the maximum iteration are 100, 1,000, and 1,000 respectively. The operator probability is set to 0.02 for mutation, and 0.8 for crossover in which 0.4 for one-point crossover, and 0.4 for two-point crossover.

Furthermore, for the DE-based GEP, SLGEP, Automatically Designed Function (ADF) in Zhong et al. (2015) has been removed to ensure the consistency as the AccGEP framework. The function set, terminal set, chromosome length, population size, maximum iteration should be set as the same settings as AccGEP, as aforesaid. In terms of the DE-based evolutionary operators introduced in section 4.2.2, the mutation factor, *F*, crossover factor, *CR*, and the mandatory index *k*, are all generated randomly according to their corresponding domain.

The original parameters of EMC-GEP only involve the archive replacement parameter, *arp*, as well as the transfer interval, δ. In this study, based on the empirical trials of the authors, *arp* and δ are set to 0.8 and 10 reciprocally for a preliminary study.

### 5.2. Experiment Data

The experiment datasets in the comparative study are mainly high-dimensional low sample size data as illustrated in Table 1, involving those datasets of which the dimension and sample size are both moderately small, thereby embodying the performance of EMC-GEP compared with the original method in diversified circumstances.

**Table 1:** Data information with dimension size, sample size, and class size.

**Index**	**Name**	**Features**	**Samples**	**Classes**
1	DLBCL-A	661	141	3
2	DLBCL-B	661	180	3
3	Armstrong-2002	2,063	62	3
4	Lapointe-2004	1,625	69	3
5	Alizadeh-2000	2,116	72	4
6	Wine	13	178	3
7	Lung Cancer	56	32	3
8	Urban Land Cover	148	675	9
9	TOX-171	5,748	171	4
10	GLA-BRA-180	49,151	180	4

Among these datasets, Urban Land Cover (Johnson and Xie, 2013) is a categorization dataset for image information, and Wine (Aeberhard et al., 1992) is a widely used multi-classification dataset. Moreover, we also adopt some bio-information data involving DLBCL-A (Hoshida et al., 2007), DLBCL-B (Hoshida et al., 2007), and Lung Cancer (Hong and Yang, 1991). Complex gene expression data, encompassing Alizadeh-2000 (Alizadeh et al., 2000), Lapointe-2004 (Lapointe et al., 2004), Armstrong-2002 (Armstrong et al., 2001), TOX-171 (Kwon et al., 2012), and GLA-BRA-180 (Sun et al., 2006), are employed as well for a more comprehensive comparison. In this article, for each dataset, 75% of data serves as training data, while 25% of data serves as testing data.

### 5.3. Comparison Results

#### 5.3.1. Comparison With AccGEP

As depicted in Table 2, five methods are utilized to analyze the 10 datasets to give a brief intuition about the performance of each strategy. For AccGEP-GA, GEP with GA operator (i.e., mutation operator and crossover operator as discussed above) is implemented under AccGEP framework. Accordingly, EMCGEP-GA is based on the AccGEP-GA with additional knowledge transfer for crossover. On the other hand, AccGEP-DE is the implementation of GEP with DE operator (i.e., “Current-to-Best” and “Frequently-based Sampling”) under the AccGEP framework. More precisely, EMCGEP-DE1 is based on AccGEP-DE with additional knowledge transfer for “Current-to-Best,” while EMCGEP-DE2 is grounded on EMCGEP-DE1 with extensive knowledge transfer for “Frequently-based Sampling.”

**Table 2:** Accuracy comparison between AccGEP and EMCGEP under distinct operators.

**Data index**	**AccGEP-GA**	**EMCGEP-GA**	**AccGEP-DE**	**EMCGEP-DE1**	**EMCGEP-DE2**
1	72.9 (4)	71.6 (5)=	75.1 (3)	**77.4 (1)+**	75.6 (2)=
2	74.4 (4)	72.8 (5)=	78.9 (2)	**81.4 (1)+**	78.9 (2)=
3	77.2 (5)	81.1 (3)+	78.9 (4)	**83.9 (1)+**	83.3 (2)+
4	58.2 (4)	46.5 (5)−	61.7 (2)	60.6 (3)=	**62.9 (1)=**
5	53.8 (4)	**60.0 (1)+**	56.3 (3)	55.0 (5)=	**60.0 (1)+**
6	94.9 (2)	86.8 (5)−	90.4 (4)	93.2 (3)+	**95.0 (1)+**
7	48.8 (2)	47.5 (4)=	48.8 (2)	40.0 (5)−	**52.5 (1)+**
8	74.4 (2)	**75.0 (1)=**	72.2 (5)	74.1 (3)+	73.8 (4)+
9	50.0 (4)	48.6 (5)=	55.6 (2)	**56.7 (1)=**	52.6 (3)−
10	58.9 (5)	59.3 (4)=	63.7 (2)	63.1 (3)=	**64.7 (1)=**
Average rank	3.6	3.8	2.9	2.6	1.8

*The bold values stand for the best performance across all the methods upon a given dataset*.

In Table 2, the fundamental data is the accuracy of the multi-classifier in percentage, and the rank number is included in the parenthese to give the relative order for performance of these methods, to supply a brief intuition of the comparison. As for the significance test, “+,” “=,” “−,” represent our method is significantly better than the original method, has no significant difference with the original method, and is significantly worse than the original method, with Wilcoxon sign-rank test (Wilcoxon, 1992) at α = 0.1. To clarify, the test for EMCGEP-GA is conducted to compare with AccGEP-GA, and the tests for EMCGEP-DE1 and EMCGEP-DE2 are conducted to compare with AccGEP-DE. In this way, the effectiveness of knowledge transfer on each component can be clearly investigated.

At length, for knowledge transfer on canonical GEP operators, there is no significant difference between AccGEP-GA and EMCGEP-GA. Even for the average rank among those five algorithms, AccGEP-GA and EMCGEP-GA share the similar rank number. This result can be attributed to the ambiguous structure of GEP. Albeit in GP-based knowledge transfer study, the segments of the expression tree serve as the useful structure to different problems, the knowledge transfer of GEP string segments is in a higher level. Since the active structure is the expression tree, the transfer upon the encoding string tends to be more indirect and more ambiguous. Hence, considering two best results in Table 2, although the idea of “abstract knowledge transfer” is intuitively promising, the algorithmic details still require more careful designs. For instance, in each evaluation of GEP individual, a great portion of the string may be the inactive area during decoding, thus the segment-based knowledge transfer somehow may be a cost of time resources, and then it is no wonder why the transfer process cannot enhance the classification accuracy in limited evaluations.

Conversely, the knowledge transfer on DE-based operators basically can attain significantly better results compared with AccGEP-DE. Notably, the average rank of DE-based GEP is apparently better than canonical GEP. Moreover, the average ranks of EMCGEP are also better than the baseline method AccGEP-DE. To elaborate the results, the knowledge transfer upon “Current-to-Best” can possibly lead to the exploration toward the valuable operator in other binary classification of the GEP population, thereby avoiding lasting reliance on mutation operators when stuck in local minima. To be specific, instead of transferring knowledge by the segment structure in EMCGEP-GA, the basic transfer ingredient in EMCGEP-DE is gene, which can more efficiently change the solution structure. Since when the target position in GEP individual is active, then a new injected gene can hopefully change the whole structure of the original individual, which can explore the searching space effectively when the evolution process is stuck in the local minima. Grounded on EMCGEP-DE1, the knowledge transfer on feature, “Frequently-based Sampling,” highly depends on the problem dimension. For those datasets with extremely high dimension like gene expression data, data 3 and data 10, transfer on feature to some extent will make no difference due to the complex distribution and the limited evaluations. But according to its average rank (1.8) compared with that of EMCGEP-DE1 (2.6), the feature transfer is still a promising avenue for knowledge transfer mechanism if adopting more detailed rules and employing more well-allocated computational resources.

#### 5.3.2. Comparison With Other Classifiers

In Table 3, EMCGEP-DE1, EMCGEP-DE2 are employed to compare with DT and KNN under the given datasets. Specifically, DT and KNN are implemented with scikit-learn (Pedregosa et al., 2011) in python with default settings.

**Table 3:** Accuracy comparison with DT, KNN, and EMCGEP under distinct operators.

**Data index**	**Decision tree**	**K Nearest neignbor**	**EMCGEP-DE1**	**EMCGEP-DE2**
1	76.0 (2)	**87.2 (1)**	77.4 (3)	75.6 (4)
2	75.8 (4)	**83.2 (1)**	81.4 (2)	78.9 (3)
3	80.3 (4)	**88.8 (1)**	83.9 (2)	83.3 (3)
4	**70.8 (1)**	66.3 (2)	60.6 (4)	62.9 (3)
5	74.1 (2)	**85.1 (1)**	55.0 (4)	60.0 (3)
6	93.7 (2)	68.4 (4)	93.2 (3)	**95.0 (1)**
7	45.0 (3)	**61.5 (1)**	40.0 (4)	52.5 (2)
8	**76.9 (1)**	44.4 (4)	74.1 (2)	73.8 (3)
9	56.9 (2)	**62.9 (1)**	56.7 (3)	52.6 (4)
10	58.7 (4)	**71.0 (1)**	63.1 (3)	64.7 (2)
Average rank	2.5	1.7	3.0	2.8

*The bold values stand for the best performance across all the methods upon a given dataset*.

As depicted in Table 3, although the Evolutionary Multitasking paradigm can enhance the performance of the existing AccGEP that searches rules based on evolutionary algorithms, the performance of the proposed EMCGEP is still limited compared with DT and KNN. Specifically, the classification results of DT are comparable with those of EMCGEP-DE1 and EMCGEP-DE2. Since DT and EMCGEP are both designed to construct rules according to given data in a non-parametric fashion, the behavior of these methods seem similar. However, KNN generally outperforms the proposed EMCGEP. One of the significant causes can be the intrinsic problem in GP methods, that the rule construction tend to be complex and unstable under high dimension scenario. Although GEP can alleviate the bloating issue of GP to some extent, the “evolutionary” behavior still makes the method unstable and even random. Nevertheless, like in data 6 and data 8, KNN sometimes is also unreliable confronting with data with certain distribution compared with EMCGEP.

### 5.4. Further Discussion

Grounded on the experiment results above, EMCGEP-DE1 and EMCGEP-DE2 can attain significantly better performance compared with the baseline method. Therefore, to provide a deeper insight into the working mechanism of knowledge transfer upon the “Current-to-Best” operator and the “Frequently-based Assignment” operator, this section tries to offer a more comprehensive and detailed discussion for the factors contributing to the higher-quality solutions.

**Figure 5:**
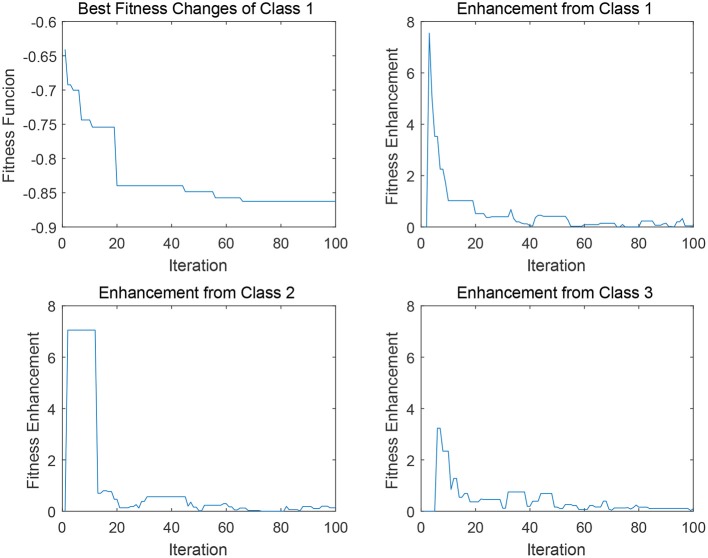
Degrees of assistance to class 1 from various “source domains” in EMC-GEP-DE1.

**Figure 6:**
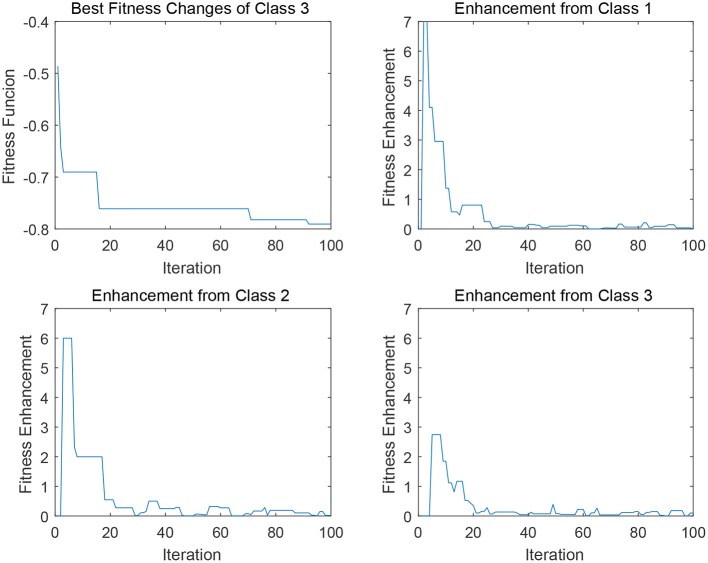
Degrees of assistance to class 3 from various “source domains” in EMC-GEP-DE2.

In Figures 5, 6, four sub-figures are given to illustrate a specific evolution process of a binary classification problem a given class in data 2 (i.e., DLBCL-B). To be specific, the first sub-figure depicts the evolution of the best individual in the binary classification population. The rest sub-figures illustrate the fitness enhancement from the archive *A*_*j*_ of each class. To clarify, the fitness enhancement here indicates the improvement of the fitness value of population *POP*_*i*_ after the transfer process Transfer (*POP*_*i*_, *A*_*j*_), which can be also concerned as the assistance from archive *A*_*j*_ of class *j* to the population *POP*_*i*_. Since the transfer interval δ is configured as 10 and the maximum iteration is 1,000, the maximum visible iteration of these sub-figures is 100.

In Figure 5, for data 2 (i.e., DLBCL-B), the best fitness variation process of the first binary classifier of class 1 is provided, along with the fitness enhancement from each source archive *A*_*j*_ in each transfer iteration. In the first sub-figure, a relatively good behavior of the convergence tendency of the class-1 binary classification is indicated, since the evolution can achieve the stepwise decrease in the fitness value so as to avoid the local optima. Generally, the self transfer process (i.e., *POP*_*i*_ ← *A*_*i*_), can imitate the process of the self evolution of the given population, (i.e., *POP*_*i*_ ← *POP*_*i*_), and the archive from class 1 does offer relatively stable transfer performance in the first several iterations as well as the continuous enhancement in the last 30 iteration. Furthermore, as depicted in the Figure 5, compared to the class 1, archive of class 2 can also supply a satisfying improvement in early 20 iterations, and the archive of class 3 can offer a stable support from iteration 20 to iteration 60 to help the target population get higher-quality solution when trapped in the local optima, thereby potentially enhancing the performance of the binary classifiers in *POP*_1_. Hence, in EMCGEP-DE1, it can be concluded that the transfer operation upon the operator “Current-to-Best” is capable of achieving performance enhancement by self transfer process, *POP*_*i*_ ← *A*_*i*_, and the cross transfer process, *POP*_*i*_ ← *A*_*j*_.

Similarly, as for the transfer operation upon the “Frequency-based Sampling,” the strategy also can offer a satisfying convergence trend as depicted in Figure 6. There are several stepwise fitness improvements for the convergence curve in the first sub-figure in Figure 6. In the very first improvement in iteration 18, three archives can offer similar support considering the fitness enhancement from each class. Whereas, in terms of the improvement in iteration 70, the abrupt change in class 2 and class 1 played the predominant factors for helping binary classifiers of class 3 to escape from the local minima. This change elaborates our assumption that, transfer process can possibly enhance the original self evolution phase. It is notable that, in this scenario, the cross transfer of class 1 and class 2 both can offer more effective and stable fitness enhancement compared to the self transfer from class 3, which indicates the promising potential of knowledge transfer for multi-classification. However, the limit of Evolutionary Multitasking is also clear from the discussions above. Since it is uncertain which knowledge source is more beneficial for the current target population according to the unstable scale of fitness enhancement illustrated in Figures 5, 6, so that it is hard to design elaborated and accurate algorithm for the given problems by Evolutionary Multitasking.

## 6. Conclusion

In this paper, knowledge transfer strategies upon canonical GEP operator and DE-based GEP operator are employed to alleviate the output conflict for multi-classification problem. In the proposed framework, a stepwise transfer is adopted to enable the segment-based transfer, DE-based transfer, as well as the feature transfer. The comparison results indicate that the DE-based transfer along with feature transfer generally can obtain significantly better performance compared to the baseline methods. Albeit the segment-based transfer for canonical GEP in this study can make no difference, some of the results and attributes of segment-based transfer still can make it special and promising, so that we concluded that this high-level transfer mechanism still require more algorithmic concern in detail. Although it is believed that knowledge transfer can enhance the existing multi-classifier, the Evolutionary Multitasking cannot tackle the intrinsic drawbacks like the randomness of the evolutionary classifiers. Furthermore, it is hard to capture the exact behavior of knowledge transfer for the evolution process, which makes it hard to design an elaborated and precise algorithm pipeline. Hence, it is deemed that both limits of Evolutionary Multitasking remain to be investigated and entails further discussion.

## Data Availability Statement

The raw data supporting the conclusions of this manuscript will be made available by the authors, without undue reservation, to any qualified researcher.

## Author Contributions

TW and JZ contributed to the design of the study, discussions of the results, writing, and reviewing of the manuscript. TW performed the experiments and wrote the main manuscript text.

### Conflict of Interest

The authors declare that the research was conducted in the absence of any commercial or financial relationships that could be construed as a potential conflict of interest.
